# Immunoadjuvant Properties of the Rho Activating Factor CNF1 in Prophylactic and Curative Vaccination against *Leishmania infantum*

**DOI:** 10.1371/journal.pone.0156363

**Published:** 2016-06-03

**Authors:** Grégory Michel, Bernard Ferrua, Patrick Munro, Laurent Boyer, Nassim Mathal, Daniel Gillet, Pierre Marty, Emmanuel Lemichez

**Affiliations:** 1 Inserm U1065, Centre Méditerranéen de Médecine Moléculaire, Team “Microbial toxins in host pathogen interactions”, Equipe labellisée ligue contre le cancer, Nice, France; 2 Université de Nice-Sophia Antipolis, Faculté de Médecine, Nice, France; 3 Centre Hospitalier Universitaire de Nice, Laboratoire de Parasitologie-Mycologie, Nice, France; 4 CEA, iBiTecS, SIMOPRO, Paris Saclay University, LabEx LERMIT, Gif sur Yvette, France; Institute Pasteur, FRANCE

## Abstract

There is a need to develop new effective immunoadjuvants for prophylactic or therapeutic vaccines against intracellular pathogens. The activation of Rho GTPases by bacterial cytotoxic necrotizing factor 1 (CNF1) elicits humoral protective responses against protein antigens. Here, we set out to investigate whether CNF1 activity initiates humoral immunity against co-administered parasite antigens and anti-microbial immune signaling. We report that co-administration of wild-type (WT) CNF1 with *Leishmania* (*L*.) promastigote antigens at the nasal mucosa triggered prophylactic and curative vaccine responses against this parasite. Vaccination of the mucosa with promastigote lysate antigens combined with WT CNF1 conferred protection against high inoculum *L*. *infantum* infection, which reached 82% in the spleen. Immune parameter analysis by antigen recall indicated robust T-helper (Th)1 polarization of immune memory cells, with high IL-2 and IFN-γ production combined with decreased IL-4 production. Additionally, we explored the curative effect of WT CNF1 on previously infected animals. We observed that PL combined with WT CNF1, but not the inactive C866S mutant CNF1 (mCNF1), induced a 58% decrease in the parasite burden in the spleen.

## Introduction

The discovery of the molecular basis of innate immunity has boosted the development of vaccine adjuvants on the basis of their capacity to stimulate innate immune receptors [1]. The family of bacterial effectors catalyzing the activation of Rho proteins has attracted growing attention because of their capacity to stimulate the immune system [2–6]. It has now been established in different model systems that cells perceive robust activation of Rho GTPases by virulence factors as a danger signal that is translated into effective anti-bacterial immune responses [5–7]. Here, we address whether a microbial effector targeting Rho GTPases can be translated into an adjuvant for vaccination against *Leishmania infantum*.

Host Rho GTPases are essential elements in host-pathogen interactions. The cytotoxic necrotizing factor-1 (CNF1) is an A-B toxin produced by uropathogenic strains of *Escherichia coli*. Wild-type (WT) CNF1 specifically deamidates the glutamine 61 in Rac1/Cdc42 (Q63 in RhoA) into a glutamic acid [8–10]. WT CNF1, but not the catalytically inactive mutant C866S of CNF1 (mCNF1), catalyzes the activation of small Rho GTPases [9,10]. Consequently, WT CNF1 can be used to increase the flux of activated Rho GTPases in host cells and the downstream Rho GTPase signaling pathways [11]. Although the small GTPases of the Rho protein family are frequent targets of post-translational modifications that are catalyzed by bacterial toxins, they contribute to sensing bacterial virulence [6,12]. Genome-wide gene expression analysis in cells treated with WT CNF1 has revealed the induction of a large panel of NF-κB-driven pro-inflammatory cytokines and chemokines [2]. More recent studies have begun to underscore the importance of the RIPK/NF- κB and ASC/Caspase-1 signaling axis in host anti-bacterial responses modulated by WT CNF1 and the Rac GTPases [5–7,13]. It is important to determine the spectrum of pathogens for which the CNF1 activity can be exploited to develop vaccines.

*Leishmania infantum/chagasi* is the causative agent of visceral leishmaniasis (VL), which is endemic in numerous southern countries, notably in the Mediterranean basin [14,15]. VL is fatal if left untreated and represents the second most challenging infectious disease worldwide [15]. Hence, part of the human population is chronically affected by poorly understood health consequences. Apart from humans, dogs are the main victims and reservoir. The current treatments are based on antibiotherapy and have serious limitations, such as high costs and toxicity [16]. For these reasons, and on the basis of the robust immunity to reinfection observed in cured patients, several vaccine trials against VL have been performed [17]. *Leishmania* parasites harness phagocytic cells, notably monocytes, in order to survive and replicate. Clinical studies of VL have suggested that decreased T-helper (Th)1 and increased Th2 responses are the hallmarks of the disease [15]. Hence, treatments that actively increase Th1 immune responses can promote the clearance of the parasites [18].

CNF1 activity and the downstream activation of Rac are sufficient to promote efficient host immune responses against bacteria [7,13]. We have begun to divert this toxin’s Rho activating property in the development of an immunoadjuvant for mucosal vaccination. We established that CNF1 activity stimulates the systemic and mucosal production of IgG and IgA antibodies against ovalbumin and tetanus toxoid [2,3]. Mice immunized against tetanus toxoid together with WT CNF1 show specific and long-lasting protection against a challenge by 10-fold of the LD_50_ of tetanus toxin [4]. It is now of interest to determine whether WT CNF1 can also stimulate Th-1 cellular immunity against an intracellular pathogen.

## Materials and Methods

### Mice and ethics statement

The protocol was approved by the Committee on the Ethics of Animal Experiments of the School of Medicine of the University of Nice, France (Permit Number: 2010–45). Groups of BALB/c female mice were purchased from Charles River at 6 weeks of age (Le Genest St. Isle, France). The mice were maintained and handled according to the regulations of the European Union and the French Ministry of Agriculture as well as to the FELASA (the Federation of Laboratory Animal Science Associations) recommendations. All efforts were made to minimize or avoid suffering.

### *L*. *infantum* parasites, antigens and CNF1

*L*. *infantum* MON-1 (MHOM/FR/94/LPN101) was isolated from a patient with Mediterranean visceral *Leishmania* that was contracted in Nice, France. *L*. *infantum* promastigotes were routinely grown at 26°C in Schneider’s medium, as previously described [19]. *L*. *infantum* clones encoding firefly luciferase were generated as previously described [20].

For the promastigote lysate (PL) preparation, stationary phase *L*. *infantum* promastigotes were washed and suspended at 10^9^/ml in distilled water [19]. The suspension was submitted to 5 freeze/thaw cycles to generate PL. Typically, 5 mg of *Leishmania* protein was obtained from 10^9^ parasites.

Recombinant wild-type CNF-1 (WT CNF1) and its catalytically inactive form (CNF1-C866S; mCNF1) were produced and purified as previously reported [21]. Both recombinant proteins were passed through a polymixin B column (Affinity pack TM-detoxy gel TM, Pierce), and the lack of endotoxin content was verified using a colorimetric LAL assay (LAL QCL-1000, Cambrex). Each CNF1 preparation stock (2 mg/ml) was shown to contain less than 0.5 endotoxin units/ml.

### Endonasal immunization and challenge in BALB/c mice

Groups of 7 mice were immunized 3 times at 2-weeks intervals with 15 μg of PL together with 1 μg WT CNF1 or 1 μg catalytically inactive CNF1 C866S (mCNF1). PL preparations were delivered into the nasal mucosa with a micropipette in 10 μl volumes of Dulbecco’s phosphate-buffered saline (PBS, from Gibco life technologies) (5 μl per nostril). Fourteen days after the last boost, mice were challenged via the intraperitoneal route with 10^8^ stationary phase *L*. *infantum* metacyclic parasites. One month later, the mice were sacrificed, and spleen section were collected and analyzed for parasite content by ELISA sandwich technique [22]. Briefly, parasite antigens in infected tissues were extracted with Nonidet-P40 detergent and were captured by anti-*L*. *infantum* human IgG that were insolubilized onto microtiter plate and were subsequently revealed using anti-*L*. *infantum* F(ab)' fragments labelled with peroxidase.

### Analysis of vaccine-induced immune responses

To assess total IgG titers, blood samples were recovered from the tail vein after vaccination (one day before infection) and before mouse dissection (one month after infection). IgG antibody responses were assessed at a 1/100 dilution by ELISA using PL-coated plates, as reported [19]. Vaccine-induced cellular immunity was measured post-vaccination using *in vitro* antigen recall experiments on spleen homogenates as follows: the spleens from each individual mouse (5 per group) were homogenized in sterile PBS, and erythrocytes were lysed at room temperature using 10 mM NaHCO_3_ containing 155 mM NH_4_Cl and 0.1 mM EDTA. Splenocytes were then washed twice with PBS, counted and suspended at 5×10^6^ cells/ml in DMEM containing 2 mM glutamine, 1 mM sodium pyruvate, 100 U/ml penicillin, 100 μg/ml streptomycin, 50 μM 2-mercaptoethanol and 10% fetal calf serum. Cell suspensions were cultured for 48 h in the presence or absence of 50 μg/ml of PL. Supernatants were harvested and assayed for IL-2, IL-4 and IFN-γ content by indirect sandwich ELISA (Pharmingen, Clinisciences). The threshold sensitivities of the techniques were in the range of 20–30 pg/ml.

### Statistical analysis

Non-parametric Mann-Whitney tests were performed using GraphPad Prism version 5.0d for Mac (GraphPad Software, San Diego California USA, www.graphpad.com).

## Results

### CNF1 activity stimulates humoral IgG responses against *L*. *infantum* antigens

**Fig 1:**
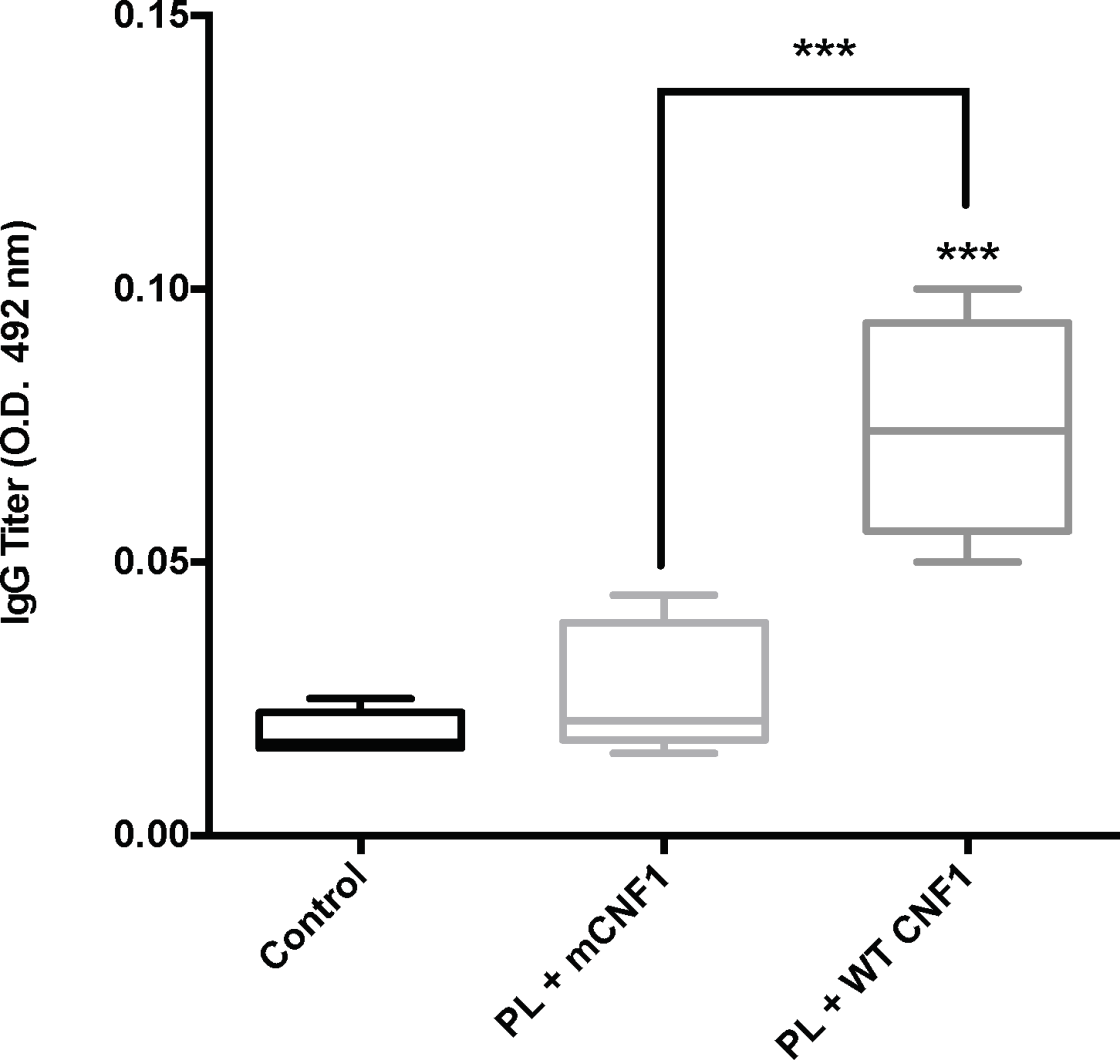
Antibody responses to *L*. *infantum* antigens post-vaccination. The anti-PL IgG antibody responses were measured post-vaccination by ELISA (one day before infection). Mice were immunized intranasally with 3x15 μg promastigote lysate (PL) plus either wild-type CNF1 (PL + WT CNF1) or catalytically inactive CNF1 (PL + mCNF1). The controls represent infected but non-immunized animals. Serum samples were tested at a 1/100 dilution and evaluated using HRP-labeled anti-mouse IgG. The interquartile ranges as well as the 10–90% percentiles are presented for each group. ***: p<0.001. The results are representative of 2 independent experiments. n = 7.

In this study, we first sought to determine the efficacy of WT CNF1 as a specific immunoadjuvant for the induction of protective responses against an intracellular pathogen. Additionally, we sought to evaluate the efficacy of this adjuvant for needle-free vaccination by topical delivery through the nasal mucosa. The immuno-modulatory effects of WT CNF1 rely on its catalytic activity, with mCNF1 catalytically inactive mutant having no effect on humoral responses [3,4]. Therefore, we directly compared the effect of WT CNF1, to that of mCNF1. As infectious model, we choose mice infection with *L*. *infantum*. Mice were immunized 3 times at 2-week intervals with promastigote lysate (PL), which was supplemented with either WT CNF1 (PL + WT CNF1) or the catalytically inactive mutant CNF1-C866S (PL + mCNF1) as a control. At first, we monitored the adjuvant effect of WT CNF1 by measuring the IgG antibody titers against PL in the sera. Under these conditions, we observed a modest but reproducible 4-fold increase in the serum IgG-titer of PL + WT CNF1 immunized mice (Fig 1). We concluded that CNF1 activity enhances immune responses against *L*. *infantum* antigens.

**Fig 2:**
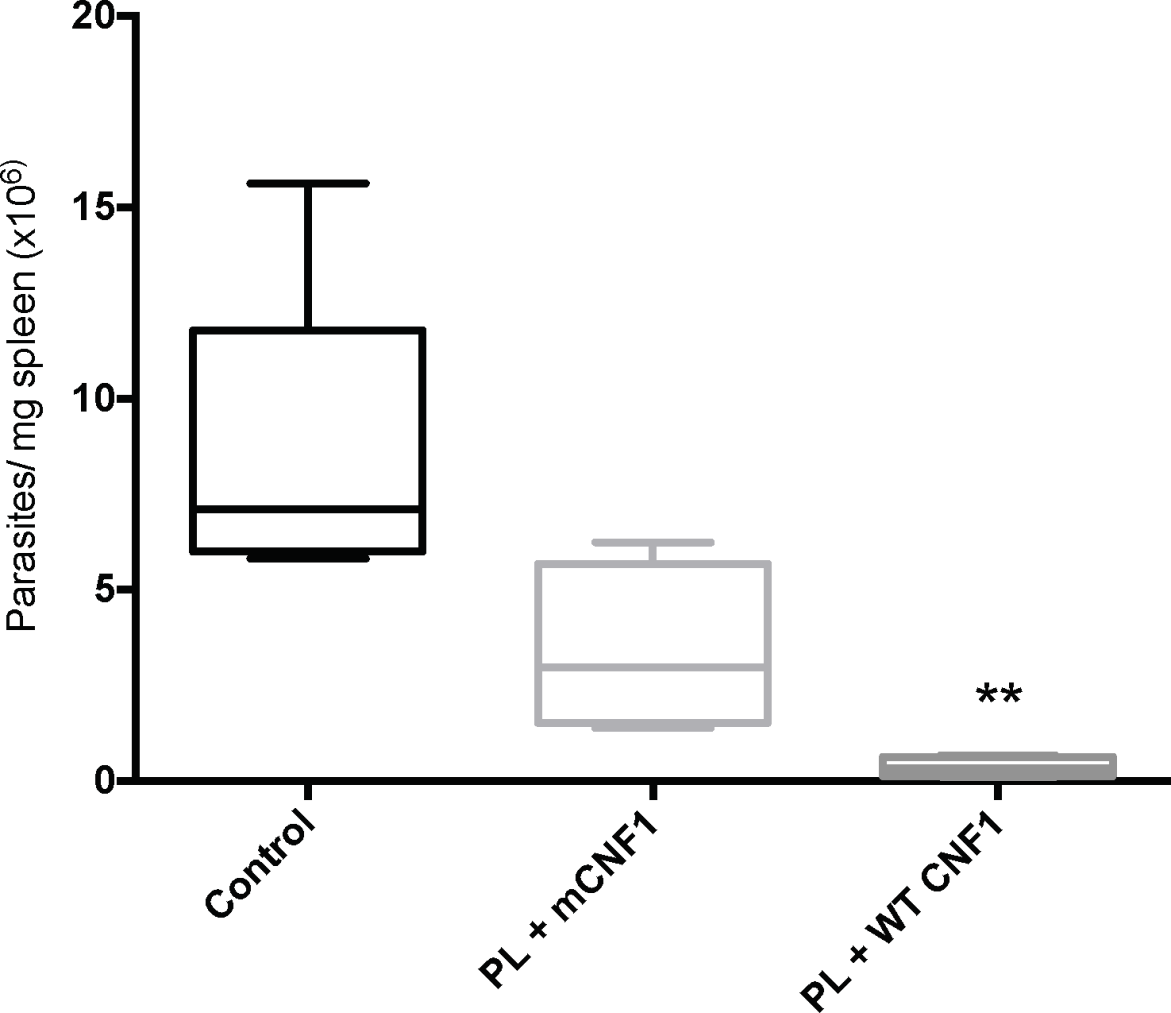
Protective effects of nasal immunizations against *L*. *infantum* infection. BALB/c mice were immunized with promastigote lysate plus either wild-type CNF1 (PL + WT CNF1) or catalytically inactive CNF1 (PL + mCNF1). Fourteen days after the last boost, the mice were intraperitoneally challenged with 10^8^ stationary phase *L*. *infantum* metacyclic parasites. The controls represent infected but non-immunized animals. Spleen parasite burdens were quantified 1 month later by ELISA. The bars indicate the mean parasite loads ± SEM. **: p<0.01. The results are representative of 2 independent experiments. n = 7.

We then established the extent of protection against *L*. *infantum* in animals under different immunization conditions. Groups of 7 mice were immunized with PL supplemented with either WT CNF1 (PL + WT CNF1) or mCNF1 (PL + mCNF1), prior to infection with high loads of 10^8^ infective metacyclic parasites. Mice were sacrificed one month later to analyze the parasite content in the spleen (Fig 2). In the naïve group, we measured a typical parasite burden ranging from 6-15x10^6^ parasites/spleen; mean = 8.5x10^6^ (Fig 2). The live parasite level dramatically decreased by 23-fold in mice that were immunized with PL together with WT CNF1 compared with controls. To evaluate the effects of CNF1 activity, we also quantified the parasite levels in a group of mice immunized with PL + mCNF1. The results revealed that the WT CNF1 catalytic activity produced a marked 6-fold increase of protection compared with mCNF1. Together, these experiments suggest that mice immunized against PL together with active WT CNF1 develop a strong resistance to infection.

**Fig 3:**
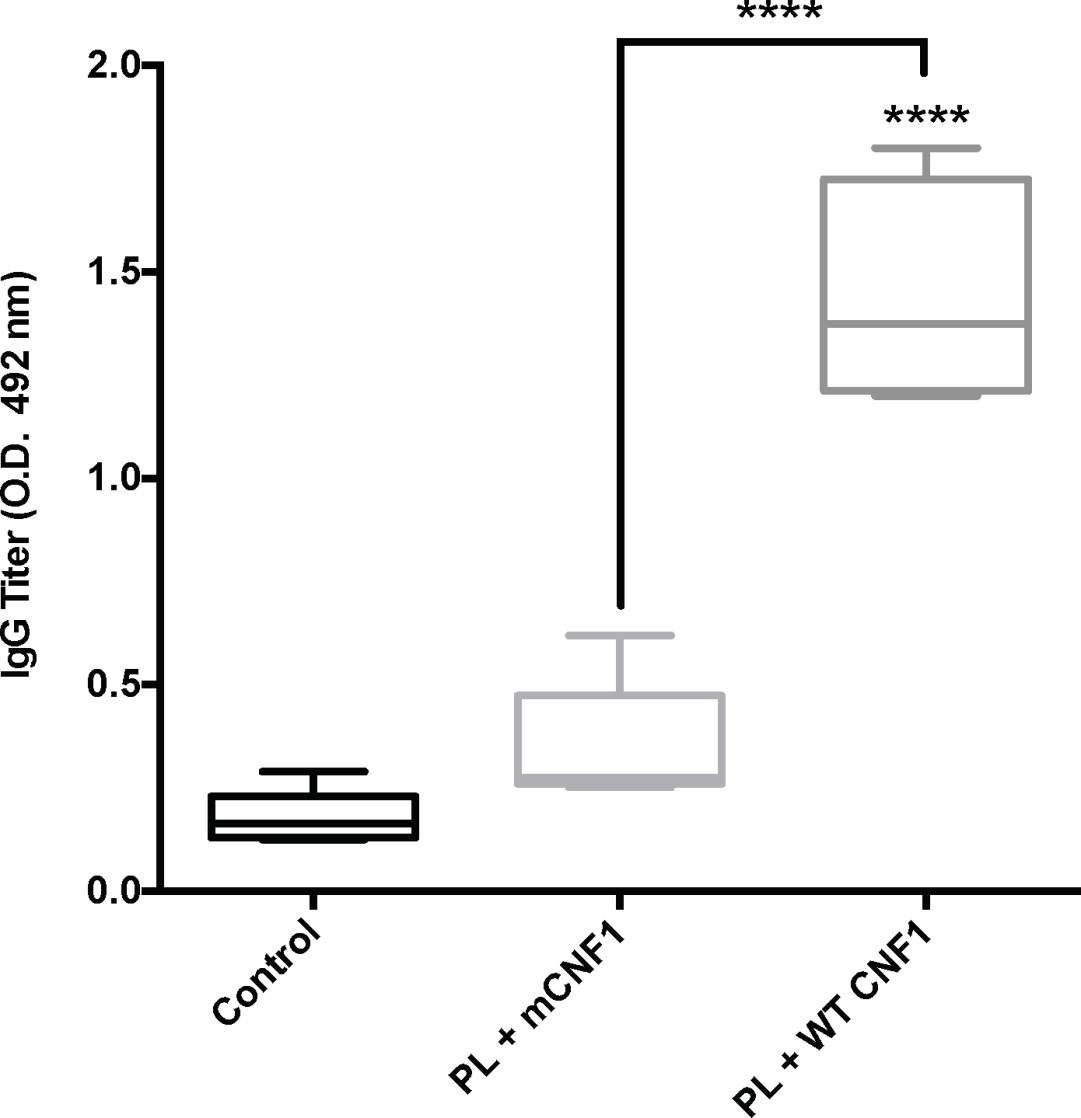
Antibody responses to *L*. *infantum* antigens post-infection. Anti-PL IgG antibody responses measured by ELISA post-infection in vaccinated mice. Mice were immunized intranasally with 3x15 μg promastigote lysate plus either wild-type CNF1 (PL + WT CNF1) or catalytically inactive CNF1 (PL + mCNF1). Serum samples were collected one month after infection and tested at a 1/100 dilution and were evaluated using HRP-labeled anti mouse IgG. The interquartile ranges as well as the 10–90% percentiles are presented for each group. ***: p<0.001. The results are representative of 2 independent experiments. n = 7.

In parallel, we assessed the IgG-titer against PL in infected mice (Fig 3). The results showed a 3-fold increase in the IgG level in PL + WT CNF1 immunized mice compared with naïve and PL + mCNF1 conditions (Fig 3). Our data are in good agreement with our findings that active CNF1 together with promastigote lysate conferred a high resistance to infection in vaccinated mice.

### WT CNF1 primes T-cell stimulatory responses against *L*. *infantum*

**Fig 4:**
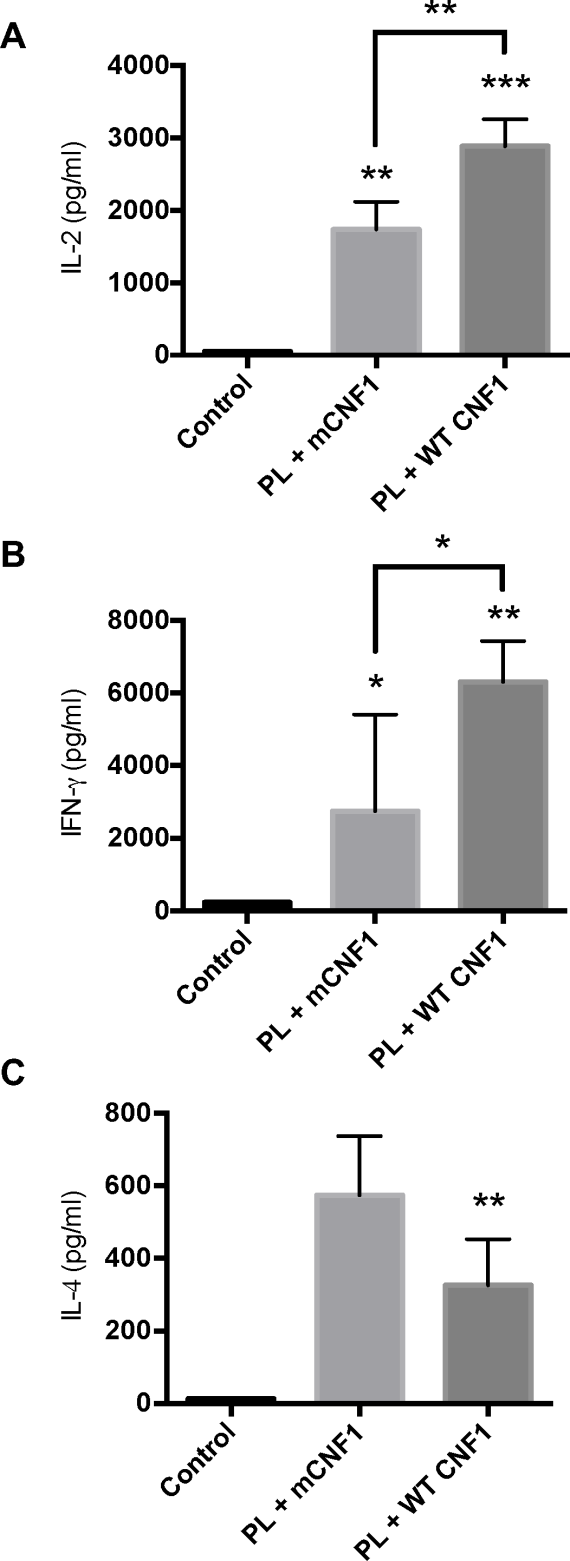
*In vitro* antigen recall experiments. Spleen homogenates from mice immunized via the nasal route with promastigote lysate (PL) plus either wild-type CNF1 (PL + WT CNF1) or catalytically inactive CNF1 (PL + mCNF1) and infected with 10^8^ stationary phase *L*. *infantum* metacyclic parasites were challenged with 50 μg/ml PL for 48 hours. The supernatants were collected and assayed for IL-2 (A), IFN-γ (B) and IL-4 (C) by ELISA. The bars represent the mean cytokine production ± SEM. *: p<0.05, **: p<0,01, ***: p<0,001. n = 7.

Th1 cellular immune responses confer animals and humans with a capacity to control *Leishmania* multiplication and dissemination [23,24]. We investigated whether WT CNF1 might stimulate T-helper Th1 responses. This was assessed in isolated splenocytes by means of antigen recall. Fig 4 shows IL-2, IFN-γ and IL-4 production levels recorded after PL-antigen recall. No cytokine production was recorded after *in vitro* PL-antigen stimulation in naïve mice spleen cells. In contrast, robust cytokine responses were recorded in mice immunized with PL. Interestingly, these responses differed among the different immunization conditions with catalytically active or inactive CNF1. The highest IL-2 and IFN-γ memory responses to PL recall were measured in mice immunized with PL + WT CNF1 compared with PL + mCNF1 (Fig 4A and 4B). Additionally, we measured a 2-fold decrease in IL-4 production in mice immunized with a catalytic form of CNF1 (Fig 4C), which produced an IFN-γ/IL-4 ratio approximately 4-fold higher in the PL + WT CNF1 vaccinated mouse group compared with the group immunized with PL + mCNF1. This profile of immune cell responses against PL, which included an increase in IL-2 and IFN-γ combined with a decrease in IL-4, indicates that CNF1 activity stimulates pro-T-helper Th1 cellular responses. This result is in agreement with the capacity of WT CNF1 to promote protection against *L*. *infantum* infection in mice.

### WT CNF1 shows curative activity against *L*. *infantum*

**Fig 5:**
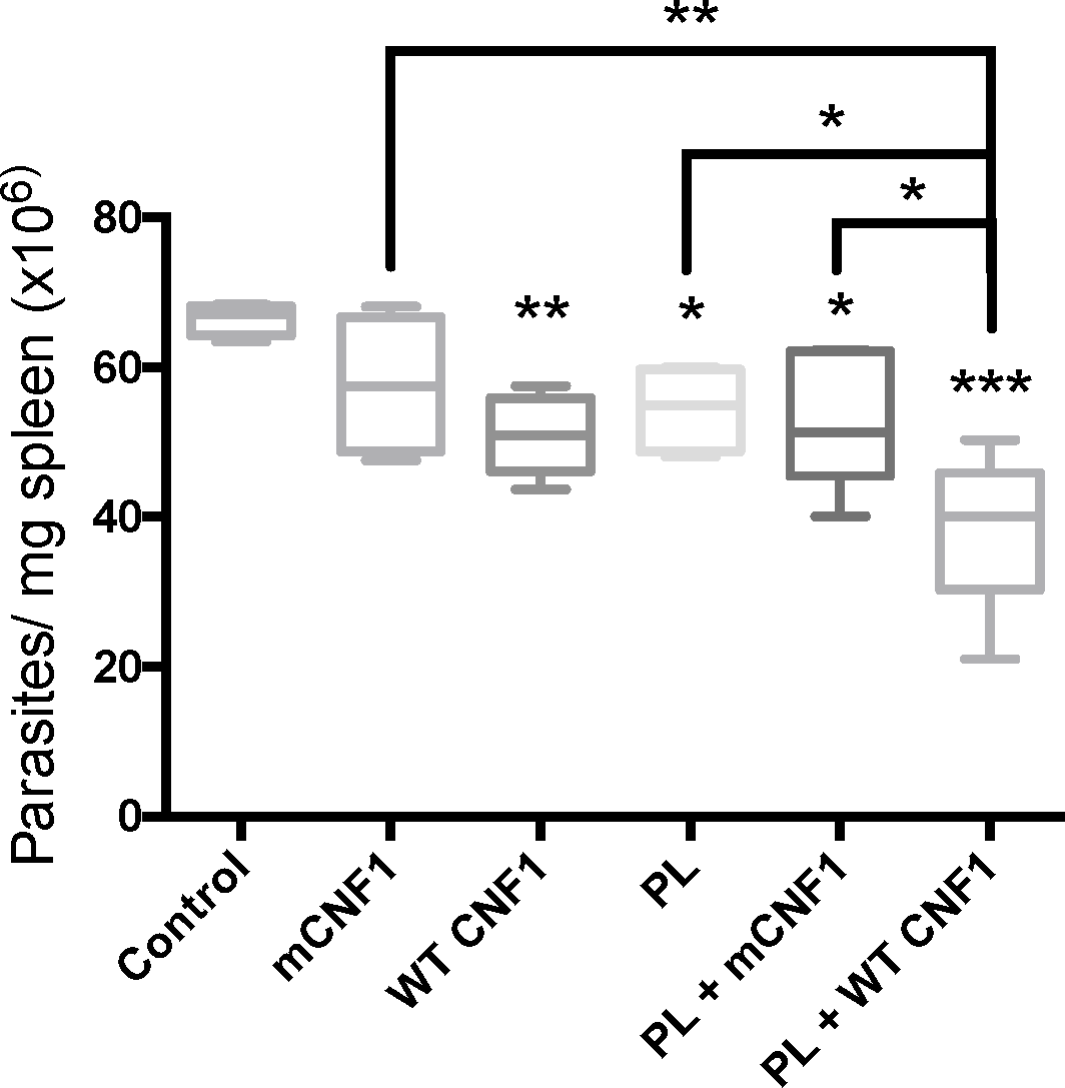
CNF1 activity confers curative immunoadjuvant properties. BALB/c mice were first infected with 3x10^6^ of stationary phase parasites. Fourteen days post-infection, mice were immunized via the nasal route with promastigote lysate (PL), wild-type CNF1 (WT CNF1), catalytically inactive CNF1 (mCNF1), PL plus either WT CNF1 (WT CNF1 + PL) or catalytically inactive CNF1 (mCNF1 + PL). The controls represent infected but non-immunized animals. Twenty-one and twenty-eight days post infection, the mice were immunized again. At day 42, the mice were sacrificed, and parasite numbers were determined by quantitative PCR using mouse spleen DNA extracts. The bars represent the mean cytokine production ± SEM. *: p<0.05, **: p<0,01, ***: p<0,001. n = 5.

**Fig 6:**
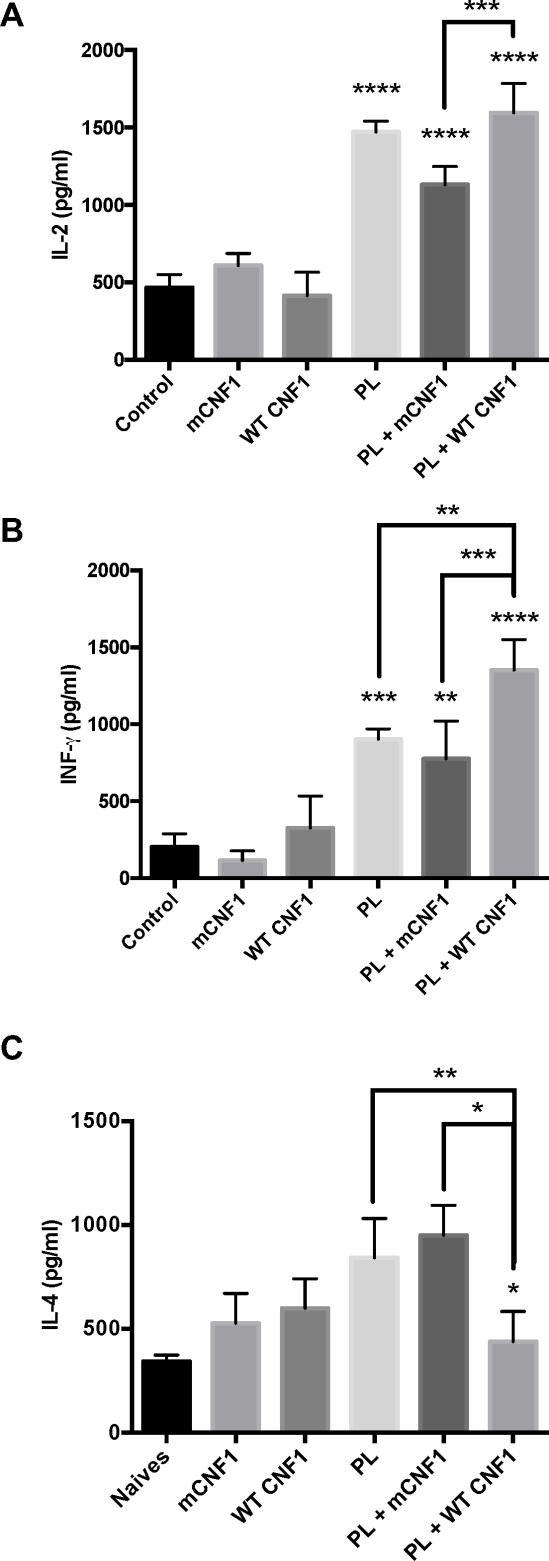
*In vitro* antigen recall for curative experiments. BALB/c mice were first infected with 3x10^6^ stationary phase parasites. Fourteen days post-infection, the mice were immunized via the nasal route with promastigote lysate (PL), wild-type CNF1 (WT CNF1), catalytically inactive CNF1 (mCNF1), or promastigote lysate plus either WT CNF1 (PL + WT CNF1) or catalytically inactive CNF1 (PL + mCNF1). Twenty-one and 28 days post infection, the mice were immunized again. The controls represent infected but non-immunized animals. At day 42, the mice were sacrificed, and spleen homogenates were challenged with 50 μg/ml PL for 48 hours. Supernatants were collected and assayed for IL-2 (A), IFN-γ (note, on graph it is INF) (B) and IL-4 (C) by ELISA. The bars represent the mean cytokine production ± SEM. *: p<0.05, *: p<0.05, **: p<0,01, ***: p<0,001. n = 5.

The above data revealed a previously unknown property of WT CNF1 in stimulating a cytokine response, thus demonstrating its strong capacity to stimulate pro T-helper Th1 immune responses. This prompted us to assess whether the CNF1 activity might also be endowed with adjuvant curative properties. Mice were first infected and later treated with or without PL in the presence or absence of either WT CNF1 or the catalytically inactive mutant, mCNF1, as a control. Therapeutic vaccination was repeated twice at one-week intervals, and the infection was monitored at day 42 post-immunization. Fig 5 depicts the parasite burden in the spleen. The infected mice treated with WT CNF1 had a reduced parasite burden compared with the mCNF1 and control mice. Second, the infected mice treated with PL alone had a significantly reduced parasite burden. Third, the PL + WT CNF1 treatment produced a maximal parasite clearance effect. Altogether, our results show a robust reduction in parasite burden triggered by PL in combination with WT CNF1. Our previous observations indicated a higher IL-2 and IFN-γ response after PL-antigen recall when mice were vaccinated with PL + WT CNF1 compared with PL + mCNF1. We tested whether the curative properties of WT CNF1, PL and PL + WT CNF1 correlated with an increase in IL-2 and IFN-γ levels after PL-antigen recall. We measured a significant increase in IL-2 and IFN-γ cytokine responses with the PL treatment but not with the WT CNF1 treatment alone, thus suggesting a role for WT CNF1 in stimulating the Th1 response initiated by PL (Fig 6A and 6B). The highest IFN-γ cytokine response level was found when PL + WT CNF1 were combined, a result consistent with maximal parasite clearance in this treatment condition (Figs 5 and 6B). Additionally, in these experiments, we measured a decreased IL-4 production of approximately 2.1-fold (Fig 6C). The ratio of IFN-γ/IL-4 was approximately 3.8-fold higher for the mice treated with PL + WT CNF1 compared with the mice immunized with PL + mCNF1 (Fig 6). Both of our curative and prophylactic vaccination settings suggest a similar mode of action for CNF1 activity, which exacerbates the Th1 cellular responses induced by PL.

## Discussion

Here, we report the use of WT CNF1 as an immunoadjuvant in a prophylactic and curative vaccination against *L*. *infantum* Infection. We linked this property of WT CNF1 to its enzymatic activity, and we provide evidence that CNF1 activity induces an immunostimulatory cytokine profile that is biased toward a Th1 response. This study suggests that Rho GTPases are targets of great value to stimulate cellular immunity against *L*. *infantum* intracellular parasite.

A limited number of vaccine trials against the visceral species, *L*. *infantum*/*chagasi*, have been reported to date [15,17,25]. Second- and third-generation vaccine candidates are based on the use of various *Leishmania* antigen preparations combined with different adjuvants [15]. Second- and third-generation vaccines using purified or recombinant *L*. *infantum* subfractions represent a feasible option for mass vaccination campaigns; however, their efficacy generally requires the co-administration of an adjuvant [15,17]. Several compounds with adjuvant properties, including cytokines, monophosphoryl lipid A, saponins, *Cryptosporidium parvum*, *Propionibacterium acnes* and Complete Freund Adjuvant have been described in vaccination trials against *L*. *infantum* [26]. However, to our knowledge, the adjuvant effect of WT CNF1, a Rho GTPase activating protein, during vaccination against *Leishmania* species has not yet been reported. In this study, we found that catalytically active CNF1 exerted a protective effect against *L*. *infantum* infection when mice were immunized in the nasal mucosa with a promastigote lysate. Notably, WT CNF1 significantly increased the resistance to *Leishmania* infection in animals despite the use of high doses of metacyclic parasites. Extending previous reports showing that vaccination with *Leishmania* antigens confers some protection in animals [27], here, we established that this protection was dramatically improved by using WT CNF1 reaching 82% protection in the spleen and 94% in liver tissues (S1 Fig).

WT CNF1 confers protection against *L*. *infantum* infection through molecular mechanisms that remain to be fully elucidated; however, they involve the toxin catalytic activity toward Rho GTPases. Here, we showed that treatment with WT CNF1 elicited specific cellular responses characterized by increased secretion of IFN-γ and IL-2 cytokines and decreased secretion of IL-4. WT CNF1 had no effect on the levels of IL-10 production after antigen recall, a down regulator of Th1 responses (not shown). IFN-γ production has a major role in eliciting anti-parasite macrophage responses, notably it induces production of H_2_O_2_ and induction of NO synthase, which are required for intracellular parasite killing. Additionally, IL-2 production and lymphoproliferation contribute to conferring cellular immunoprotection. These protective immune responses are balanced by the immunosuppressive responses triggered by the parasite. Immunosuppressive cytokines, notably IL-4, are involved in the exacerbation of infection. Although WT CNF1 had no effect on IL-10 production, we found that catalytically active CNF1 was able to decrease IL-4 production. Thus, decreased IL-4 production combined with increased IFN-γ and IL-2 production indicates that CNF1 activity enhances immune T-cell response polarization toward the Th1 phenotype. The mechanistic behind this Th1 polarization triggered by WT CNF1 requires further investigation. One possibility is that WT CNF1 directly targets the T cell compartment. A second possibility is that WT CNF1 targets other immune cells allowing them to produce signals triggering the polarization of T cells toward the Th1 phenotype. We also reveal a significant curative effect triggered by WT CNF1 during the treatment of *Leishmania* infected animals. This curative effect is greatly enhanced in the presence of *Leishmania* antigen. While the exact mechanism by which CNF1 activity confers protection against *Leishmania* remains to be uncovered, interestingly we show here that this curative effect of WT CNF1 is specifically promoted by the co-administered antigens.

Collectively, our data provide the first indication that active WT CNF1 has vaccinal properties in promoting prophylactic and curative protection against *L*. *infantum* intracellular parasites.

## Supporting Information

S1 Fig Liver Protective effects of nasal immunizations against *L*. *infantum* infection. BALB/c mice were immunized with promastigote lysate plus either wild-type CNF1 (PL + WT CNF1) or catalytically inactive CNF1 (PL + mCNF1). Fourteen days after the last boost, the mice were intraperitoneally challenged with 10^8^ stationary phase *L*. *infantum* metacyclic parasites. The controls represent infected but non-immunized animals. Liver parasite burdens were quantified 1 month later by ELISA. The bars indicate the mean parasite loads ± SEM. **: p<0.01. The results are representative of 2 independent experiments. n = 7.
(TIFF)
