# Supplementary figures and images for: Immunoadjuvant Properties of the Rho Activating Factor CNF1 in Prophylactic and Curative Vaccination against Leishmania infantum
Source: PLoS One. 2016 Jun 3;11(6):e0156363. doi: 10.1371/journal.pone.0156363 (PMC4892475; doi:10.1371/journal.pone.0156363)

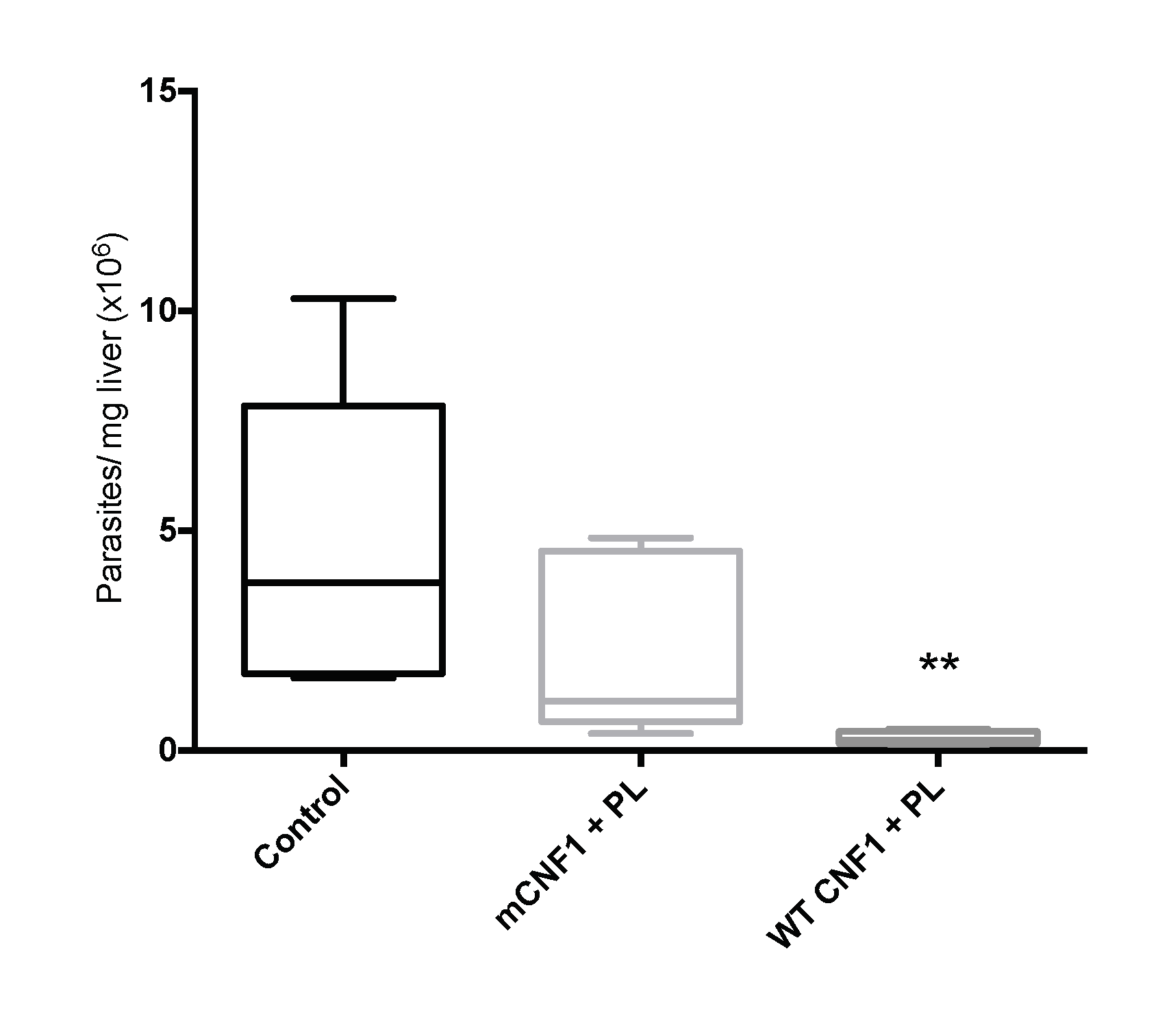

Supplement: S1 Fig — BALB/c mice were immunized with promastigote lysate plus either wild-type CNF1 (PL + WT CNF1) or catalytically inactive CNF1 (PL + mCNF1). Fourteen days after the last boost, the mice were intraperitoneally challenged with 108 stationary phase L. infantum metacyclic parasites. The controls represent infected but non-immunized animals. Liver parasite burdens were quantified 1 month later by ELISA. The bars indicate the mean parasite loads ± SEM. **: p<0.01. The results are representative of 2 independent experiments. n = 7. (TIFF) [file pone.0156363.s001.tiff]
